# Neurologic Complications of Influenza B Virus Infection in Adults, Romania

**DOI:** 10.3201/eid2304.161317

**Published:** 2017-04

**Authors:** Corneliu P. Popescu, Simin A. Florescu, Emilia Lupulescu, Mihaela Zaharia, Gratiela Tardei, Mihaela Lazar, Emanoil Ceausu, Simona M. Ruta

**Affiliations:** Carol Davila University of Medicine and Pharmacy, Bucharest, Romania (C.P. Popescu, S.A. Florescu, E. Ceausu, S.M. Ruta);; Dr. Victor Babes Clinical Hospital of Infectious and Tropical Diseases, Bucharest (C.P. Popescu, S.A. Florescu, M. Zaharia, G. Tardei, E. Ceausu);; European Society of Clinical Microbiology and Infection Study Group for Infectious Diseases of the Brain, Basel, Switzerland (C.P. Popescu, M. Zaharia);; National Institute of Research Cantacuzino, Bucharest (E. Lupulescu, M. Lazar);; Stefan S. Nicolau Institute of Virology, Bucharest (S.M. Ruta)

**Keywords:** influenza B virus, viruses, influenza, influenza vaccine, neurologic complications, meningitis/encephalitis, respiratory infections, B (Yam)-lineage clade 3, B/Phuket/3073/2013, adults, tertiary care facility, Romania

## Abstract

We characterized influenza B virus–related neurologic manifestations in an unusually high number of hospitalized adults at a tertiary care facility in Romania during the 2014–15 influenza epidemic season. Of 32 patients with a confirmed laboratory diagnosis of influenza B virus infection, neurologic complications developed in 7 adults (median age 31 years). These complications were clinically diagnosed as confirmed encephalitis (4 patients), possible encephalitis (2 patients), and cerebellar ataxia (1 patient). Two of the patients died. Virus sequencing identified influenza virus B (Yam)-lineage clade 3, which is representative of the B/Phuket/3073/2013 strain, in 4 patients. None of the patients had been vaccinated against influenza. These results suggest that influenza B virus can cause a severe clinical course and should be considered as an etiologic factor for encephalitis.

## Abstract 2

Infection with this virus should be considered as an etiologic factor for encephalitis.

Influenza viruses are negative single-stranded RNA viruses belonging to the family *Orthomyxoviridae* and cause worldwide epidemics of influenza with high rates of illness and death. Human influenza A and B viruses cause a self-limited acute respiratory infection. This infection has an abrupt onset and causes fever, chills, headache, cough, and myalgia. Every year, different strains of influenza viruses emerge because of continuous antigenic drift and interspecies gene reassortment, which cause antigenic shifts. Severe complications of influenza can involve the lower respiratory tract (pneumonia), heart (myocarditis), and central nervous system (encephalitis, myelitis, meningitis, febrile and afebrile seizures, Guillain-Barré syndrome, cerebellar ataxia) and can lead to death (*1*–*3*).

Although type A and B influenza viruses might induce neurologic complications, most published studies on virus neurotropism have focused on influenza A viruses, with an emphasis on the new A(H1N1)pdm09 virus strain after 2009 (*4**–**7*). Influenza B virus, which was isolated from a child in 1940, has steadily adapted to humans without a stable animal reservoir (*8*–*10*). The earliest report of a case of influenza B viral encephalitis was in London, UK, in 1946 (*11*), but only sporadic cases with neurologic manifestations have been reported, especially in children and adolescents. Influenza B is generally considered a mild disease with less frequent neurologic complications than influenza A (*4*–*7*).

There was major increased influenza activity in Romania during the 2014–15 influenza season: 3.5 times more cases of influenza-like illness (ILI) and acute respiratory infections than in the previous season. A total of 4,511 case-patients with ILI were reported, of which 1,709 (37.9%) were hospitalized; 3,297 (73.1%) were >14 years of age. Influenza B viruses prevailed (in 529 [56.4%] of the 938 laboratory-confirmed influenza cases), unlike the rest of Europe, where there was a predominance of type A influenza strains (*12*). We characterized influenza B virus–related neurologic manifestations diagnosed at a tertiary care facility in Romania during the 2014–15 influenza season.

## Materials and Methods

### Ethical Approval

This study was approved by the ethics committee of the Stefan S. Nicolau Institute of Virology (Bucharest, Romania). Although this was a retrospective study, informed consent was obtained from each patient included in the study, as part of routine hospital activity.

### Patients

We conducted a retrospective study of 7 patients given a diagnosis of influenza in whom neurologic complications developed. These patients were hospitalized in a tertiary care facility (Dr. Victor Babes Clinical Hospital of Infectious and Tropical Diseases, Bucharest, Romania) during the 2014–15 influenza season. 

We used the case definition for encephalitis from the 2013 Consensus Statement of the International Encephalitis Consortium. Major criterion was altered mental status (defined as decreased or altered level of consciousness, lethargy, or personality change) lasting >24 hours with no alternative cause identified. Minor criteria were fever (temperature >38°C), generalized or partial seizures, new onset of focal neurologic findings, CSF leukocyte count >5 cells/mm^3^, and abnormality of brain parenchyma on neuroimaging suggestive of encephalitis (*13*).

We collected nasopharyngeal swab specimens from all patients with ILI and sent these specimens to the National Reference Center for Influenza (Cantacuzino Institute, Bucharest, Romania) for antigenic and genetic characterization. Specimens were examined by using real-time reverse transcription PCRs (RT-PCRs) with the Superscript III Platinum One-Step Quantitative RT-PCR System (Invitrogen, Carlsbad, CA, USA) for influenza type A and type B viruses. Samples positive for influenza A viruses were tested by using a second real-time RT-PCR that discriminated between influenza A(H1N1)pdm09 and A(H3N2) virus subtypes. For samples positive for influenza B viruses, we used a second real-time RT-PCR and specific minor-groove binder probes to determine lineage (*14*).

Positive specimens were inoculated into an MDCK line, and virus isolates were characterized antigenically by using a hemagglutination inhibition assay and turkey/guinea pig erythrocytes. We used the conventional Sanger sequencing technique to monitor influenza virus evolution for the complete hemagglutinin (HA) gene. The PRISM BigDye Terminator v3.1 Ready Reaction Cycle Sequencing Kit (Applied Biosystems, Foster City, CA, USA) was used to sequence DNA templates on a PRISM 3100-Avant Genetic Analyzer (Applied Biosystems). We determined genotypes of all virus sequences from patients by alignment with sequences found in Romania with World Health Organization reference viruses. We performed phylogenic analysis by using maximum-likelihood inference and a generalized time-reversible model of nucleotide substitution and a Γ model of rate heterogeneity with RAxML version 8.00 software (*15*).

## Results

### Patients

Patient 1 was a 28-year-old woman who had uncontrolled hyperthyroidism, a 3-day history of high fever (temperature >39°C), headache, sleepiness, left upper limb motor deficit, and a Glasgow Coma Scale (GCS) score of 3−4/15. Results of cerebrospinal fluid (CSF) testing were unremarkable. The patient was intubated and mechanically ventilated after 4 hours of hospitalization.

**Figure 1:**
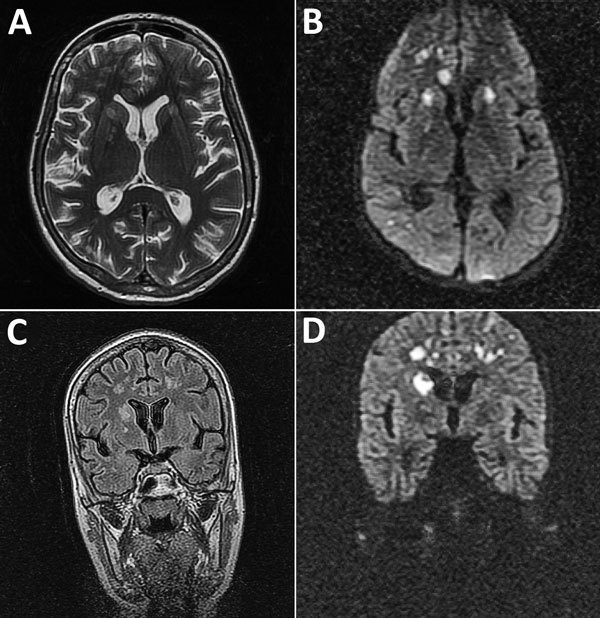
Magnetic resonance imaging of the brain of a 28-year-old woman (patient 1) who had neurologic complications of influenza B virus infection, Romania. A) Axial T2 image showing multiple areas of T2-associated hyperintense lesions with involvement of the genu corpus callosum, bilateral internal capsule, and several areas of white matter in the right frontal lobe, and more discreetly at the limit between the right parietal and occipital lobe. B) Axial diffusion-weighted image showing restricted diffusion associated with lesions. C) Coronal fluid–attenuated inversion recovery image showing multiple hyperintense lesions in the right caudate head and the cortical and deep white matter of the frontal lobes. D) Coronal diffusion-weighted image showing restricted diffusion associated with lesions.

Magnetic resonance imaging (MRI) of the brain (Figure 1) showed multiple areas of T2-associated hyperintensities associated with restricted diffusion with involvement of the genu corpus callosum bilateral internal capsule and several areas of white matter in the right frontal lobe. Hyperintensities were visible at the limit between the right parietal and occipital lobe (axial T2-associated and diffusion-weighted imaging), and multiple high-signal lesions associated with restricted diffusion were present in the right caudate nucleus head and the subcortical and deep white matter of the frontal lobes (coronal fluid–attenuated inversion recovery and diffusion-weighted imaging). Despite intensive antimicrobial drug treatment with oseltamivir, acyclovir, meropenem, mannitol, corticosteroids, and thyrozol, the patient died 7 days after admission.

Patient 2 was a previously healthy 37-year-old woman who was hospitalized after 2 days of fever, rhinorrhea, myalgia that progressed to a headache, sleepiness, photophobia, vertigo, stiff neck, and a positive Romberg sign. Results of computed tomography (CT) and analysis of CSF were unremarkable. Complete resolution occurred after 9 days of treatment with oseltamivir and mannitol.

**Figure 2:**
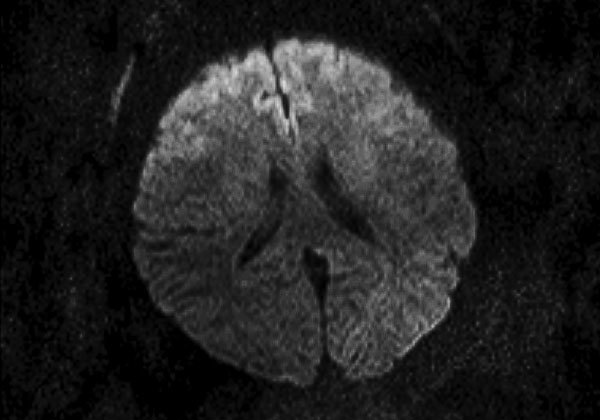
Magnetic resonance imaging of the brain of a 55-year-old woman (patient 3) who had neurologic complications of influenza B virus infection, Romania. Axial diffusion-weighted image showing restricted diffusion in the bilateral frontal cortex.

Patient 3 was a previously healthy 55-year-old woman with a history of influenza A(H3N2) virus encephalitis (in 2012). The patient was hospitalized after transfer from another clinic 10 days after onset of illness with signs and symptoms that included fever, confusion, photophobia, dizziness, right facial paralysis, aphasia, stiff neck, and coma (GCS score 8). Onset of neurologic signs occurred on day 5. Results of CT on day 7 were unremarkable. We found increased levels of proteins and cells in the CSF. Brain MRI showed symmetric diffusion restriction in the bilateral anterior frontal cortex (Figure 2). The patient was intubated and mechanically ventilated for 24 hours. She showed good progression after being given mannitol, dexamethasone, oseltamivir, and acyclovir. The patient was discharged with complete resolution after 18 days of hospitalization.

Patient 4 was a 20-year-old woman who had Russell−Silver syndrome and was being given prednisone and levothyroxine. She was admitted to an intensive care unit (ICU) already mechanically ventilated. Onset of illness was 5 days before admission and included fever, cough, and agitation. After 3 days in the hospital, nystagmus, stiff neck, and a GCS score of 9−10 were observed. Results of CSF testing and cerebral CT and MRI were unremarkable. She was given mannitol, methylprednisolone, and oseltamivir. Her condition improved after 17 days of hospitalization and was followed by complete recovery.

Patient 5 was a 57-year-old man who came to our clinic after 3 days of fever, chills, and cough. In the preceding 24 hours, headache, dysarthria, right side motor deficit, and vomiting developed. Results of CSF testing and cranial CT and MRI were unremarkable. The patient was given oseltamivir and mannitol. After 10 days, the patient was discharged, and he showed complete resolution.

Patient 6 was a 31-year-old woman who had recent breast implants. The patient was hospitalized after 4 days of fever, headache, vomiting, vertigo, photophobia, and movement and balance disorders. Results of CSF testing were unremarkable. She was given oseltamivir and mannitol, and her condition improved after 5 days.

Patient 7 was a 27-year-old woman who had influenza, fever, myalgia, headache, and vomiting 3 days before hospitalization. After 2 days, she became lethargic, had aphasia and seizures, and entered a coma; GCS score decreased to 3. Results of initial CSF testing were unremarkable. She was intubated, mechanically ventilated, and transferred to our clinic. Results of CT were unremarkable. However, MRI showed an abnormal result (multiple areas of hyperintensities).

At admission, patient 7 was comatose and intubated. She had unreactive fixed mydriasis, upward deviation of the eyes, and no corneal reflex and plantar cutaneous reflexes. Results of additional CSF testing showed pleocytosis and a high level of albumin (3.906 g/L). Renal failure and ventricular tachyarrhythmia developed. Despite treatment with oseltamivir, acyclovir, mannitol, methylprednisolone, and meropenem, the patient continued to show signs of brain death and died 3 days after admission.

### Observations

The peak of the influenza season in Romania was during February−March 2015. A total of 110 patients with ILI (90 female patients and 20 male patients) were hospitalized at the tertiary care facility during January−April 2015. The median age of patients was 43 years (range 4−93 years); only 3 patients were <18 years of age. None of the patients had been vaccinated against influenza for the current season. There were 57 patients with ILI laboratory-confirmed influenza infection: 32 patients (56.2%) were infected with an influenza B strain, 14 (24.5%) with an influenza A/H3 strain, and 11 (19.3%) with the A(H1N1)pdm09 strain. These 3 strains were present in Romania at similar prevalences throughout the influenza season.

A complicated form of influenza was diagnosed for 28 (49.1%) patients, of whom 19 (33.3%) had respiratory complications (8 infected with the A(H1N1)pdm09 strain, 6 with the A/H3 strain, and 5 with the B strain), and 9 (15.7%) had neurologic complications (8 with the B strain and 1 with the (A/H1N1)pdm09 strain). Two patients who had febrile seizures as the only neurologic manifestation were excluded from analysis (1 child with a history of febrile seizures and 1 adult with epilepsy). Eight of the 9 patients with neurologic complications were female patients. Of those infected with an influenza B strain, 7 were adults (median age 31 years, range 20−57 years), and 1 was a 4-year-old girl. Six of the 7 adult patients infected with an influenza B strain in whom neurologic manifestations developed were women.

Antiviral treatment with a neuraminidase inhibitor (oseltamivir, 75 mg 2×/d for adults and 37.5 mg 2×/d for the child) was administered to all patients upon presentation. However, most patients presented late after onset of disease, and only 1 received oseltamivir within the first 48 hours of disease onset; the other patients received antiviral treatment 72 hours after disease onset.

Demographic, clinical, imaging and laboratory data for the 7 adult patients with neurologic manifestations and laboratory-confirmed influenza B virus infection are shown in the Table. None of the patients had preexisting neurologic diseases, and the average time from disease onset to hospitalization was 4.28 days. On the basis of the case definition, 4 patients were given a diagnosis of confirmed encephalitis (major criteria and >3 minor criteria and laboratory-confirmed influenza), 2 were given a diagnosis of possible encephalitis (major criteria and 2 minor criteria), and 1 was given a diagnosis of cerebellar ataxia (no major criteria but with neurologic manifestations).

**Table:** Characteristics of 7 patients with neurologic complications of influenza B virus infection, Romania*

Characteristic	Patient						
	1	2	3	4	5	6	7
Age,y/sex	28/F	37/F	55/F	20/F	57/M	31/F	27/F
Days from illness onset	3	2	10	5	3	4	3
Medical history	Uncontrolled hyperthyroidism	Unremarkable	Influenza A(H3N2) virus encephalitis in 2012	Treated for Russell−Silver syndrome	Unremarkable	Recent breast implants	Unremarkable
Leukocyte count, cells/mm^3^	2	2	10	5	2	3	13
CSF							
Protein, g/L	0.143	0.270	2.305	0.251	0.313	0.162	7.156
Glucose, g/L	0.61	0.50	0.74	1.07	0.55	0.71	1.22
Chloride, g/L	ND	ND	7.60	6.78	7.10	6.80	7.02
Virus type	B seq EPI_ISL_179707	B seq EPI_ISL_179711	B	B	B seq EPI_ISL_182519	B	B seq EPI_ISL_182518
Cerebral imaging result	MRI, abnormal†	CT, normal	MRI, abnormal‡	MRI, normal	MRI, normal	NA	MRI, abnormal§
Diagnosis	Confirmed encephalitis	Possible encephalitis	Confirmed encephalitis	Confirmed encephalitis	Possible encephalitis	Cerebellar ataxia	Confirmed encephalitis
Length of hospitalization, d	7	9	18	17	10	5	3
Outcome	Died	Complete resolution	Complete resolution	Complete resolution	Complete resolution	Complete resolution	Died
Clinical findings	Fever, headache, sleepiness, left upper limb motor deficit, coma, GCS score 3–4	Fever, headache, sleepiness, photophobia, vertigo, stiff neck, positive Romberg sign	Fever, confusion, photophobia, dizziness, right facial paralysis, aphasia, stiff neck, coma, GCS score 8	Fever, agitation, nystagmus, stiff neck, coma, GCS score 9–10	Fever, headache, dysarthria, right side motor deficit, vomiting	Fever, headache, vomiting, vertigo, photophobia, ataxia, positive Romberg sign, movement and balance disorder	Fever, headache, vomiting, lethargy, aphasia, upward deviation of eyes, seizures, coma, GCS score 3

*All patients showed negative RT-PCR results for influenza B virus in CSF. CSF, cerebrospinal fluid; CT, computed tomography; GCS, Glasgow coma scale; MRI, magnetic resonance imaging; NA, not available; ND, not determined. †MRI on day 3. See Figure 1 for a detailed description. ‡MRI on day 8. See Figure 2 for a detailed description. §MRI on day 2 showed multiple areas of hyperintensities.

Cerebral MRI was performed for 6 patients and CT was performed for 2 patients. Abnormal brain imaging results were observed for 3 patients: changes consistent with multiple areas of hyperintensities visible in T2-associated with restricted diffusion for patients 1 and 7 (patient 1; Figure 1) and cerebral edema and diffusion restriction for patient 3 (Figure 2). Six patients were admitted to the ICU; 4 of these patients required intubation and mechanical ventilation because of neurologic complications. Two patients died, 3 and 7 days after admission. The other 5 patients showed a good outcome with complete resolution. At a follow-up 1 month after discharge, results of neurologic examinations for the 5 patients were unremarkable without any signs or symptoms during this period.

Lumbar puncture was performed for all 7 patients. Results of CSF analysis were abnormal for 3 patients (pleocytosis [>5 cells/mm^3^] and increased protein levels). All CSF samples were negative for influenza virus nucleic acids, enteroviruses, and herpes viruses.

Genetic analysis of the HA sequences of influenza B viruses isolated from nasopharyngeal swab specimens was successful for 4 patients (patients 1, 2, 5, and 7) (Table). HA phylogenetic analysis showed that all strains belonged to Yamagata-lineage clade 3, representative strain B/Phuket/3073/2013, which is distinct from the World Health Organization recommended strain included in the 2014–2015 vaccine (B/Massachusetts/2/2012–like virus).

## Discussion

We report an unusually high number of adults infected with influenza B virus who had neurologic complications at a tertiary care facility in Romania. Influenza B neurologic manifestations have been reported mainly in children. In Japan in 1998–1999, an outbreak of influenza-associated encephalitis/encephalopathy (148 cases, mostly linked with influenza A/H3N2 infection, only 17 with influenza B viruses) was observed primarily in children <5 years of age (*16*). In a 2-year National British Surveillance study, 25 cases (21 in children) of neurologic disorders were identified in patients with influenza in the United Kingdom, but only 4 of these patients (all children) were infected with influenza B virus (*17*). Eleven cases of neurologic complications in children with influenza B virus infection were reported in Taiwan in 2006 (*18*). Other cases were also reported: 1 case of influenza B virus–associated optic neuritis after meningoencephalitis in a 10-year-old boy in Italy (*19*), and 1 case of influenza B virus–associated acute necrotizing encephalopathy in a 3-year-old boy in North America (*20*).

Sporadic cases of influenza B virus with neurologic manifestations in adults have also been reported: 2 adults with influenza B virus–associated encephalopathy (*21*), 1 person with nonconvulsive epilepticus after influenza virus B infection (*22*), and 6 of 15 patients with influenza B virus–associated encephalitis (*7*). In our study, we report 7 case-patients with influenza B virus–associated neurologic complications; 2 of these case-patients died. In Romania during the 2014–15 influenza season, the mortality rate for patients with confirmed influenza B virus infections was 1.7% (*12*). In the tertiary care facility we studied, the overall mortality rate for patients infected with influenza B virus was 9.3% (3/32 confirmed case-patients), and the mortality rate for patients with influenza B virus–related neurologic complications was 28.5% (2/7 adult case-patients). All patients with neurologic complications admitted to the ICU who required intubation and mechanical ventilation did not have respiratory complications, the 2 deaths were caused by neurologic manifestations, not respiratory failure.

Although the 2014–15 influenza season in Romania was severe, older persons were less frequently infected (12.8% of ILI cases recorded were in patients >65 years of age), but hospitalization rates increased with age (from 23.7% in persons <0–1 year of age to 36.7% in persons 15–49 years of age, and 61.5% in persons >65 years of age) (*12*). The high mortality rate is not necessarily representative of the severity of influenza B virus infection because persons with severe cases are generally referred to the tertiary care facility. However, these data highlight the potentially severe progression of influenza B virus infection in adults. This severe evolution has been reported in children. In the United States in 2010–11, influenza B viruses were involved in 38% of deaths in children caused by influenza (*23*). In Japan, a 6-year national surveillance identified 50 patients (median age 4.5 years) with of influenza B virus–associated encephalopathy/encephalitis, of whom 7 (14%) died (*24*).

Most patients in our study had no previous concurrent conditions. One patient (patient 3) had a prior episode of influenza-related encephalitis with the same clinical pattern as the present infection (the previous infection was diagnosed at the same hospital in 2012). At that time, the isolated virus was identified as influenza A(H3N2) virus. This isolate was not tested for genetic mutations that might predispose a person to recurrent encephalitis. However, such infections have been reported in a family infected with a virus containing a Ran binding protein 2 mutation, which is autosomal dominantly transmitted (*17*,*25*).

In our study we isolated virus from nasopharyngeal samples; none of the CSF samples were positive for virus nucleic acids. However, influenza viruses are rarely identified in the CSF (*2*,*26*). Abnormal findings for CSF were observed in 3 patients with encephalitis who required hospitalization in the ICU (1 patient died). Two patients, both admitted to the ICU, showed major increases in protein levels in CSF, a profile usually seen in patients with severe cases (*2*,*26*,*27*). Although influenza virus nucleic acids have been detected in brain tissue, ependymal, and Purkinje cells (*28*), several studies have emphasized the role of neuroinflammation in the pathogenesis of neurologic complications mediated by high levels of proinflammatory cytokines in the CSF (cytokine storm), increased systemic inflammatory responses, or blood–brain barrier dysfunction (*29*–*34*). However, neurotropism has been investigated mainly for influenza A viruses; information on neurovirulence caused by influenza B viruses is lacking.

All isolated virus strains belonged to B(Yam)-lineage clade 3, representative strain B/Phuket/3073/2013, which belongs to a distinct antigenic cluster different from that recommended by the World Health Organization for vaccination in the Northern Hemisphere during the 2014–15 influenza season. This strain and the vaccine strain (B/Massachusetts/02/2012–like from the B/Yamagata/16/88 lineage) were cocirculating in Romania during the 2014–15 influenza season (*35*). Antigenic mismatch between a vaccine strain and a strain that prevails in a specific influenza season is common for influenza B viruses because of cocirculation of the Victoria and Yamagata lineages (*36*), and additional challenges are increased by antigenic drift of the B/Yamagata strain.

None of the patients in our study in whom neurologic complications developed were vaccinated against influenza. Vaccination coverage in Romania was extremely low; only 2.5% of the general population were vaccinated (*12*). The efficacy of the 2014–15 vaccine against influenza B virus was modest, ranging from 41% in persons 15–59 years of age to 62% in persons <14 years of age, as reported by a multicenter case–control study in the population in Europe (*37*). Some studies support the inclusion of both lineages of influenza B virus in the vaccine to reduce illness (*38*), although others suggest a more cautious approach, arguing that addition of a second influenza B virus lineage leads to a modest reduction in influenza-associated outcomes (*39*). Recently, a hedging strategy for selection of the influenza B virus lineage included in the standard trivalent vaccine has been suggested as the most effective in terms of long-term protection rates (*40*).

This study had several limitations. A cluster of severe cases is not uncommon in a tertiary care facility with an ICU, which might lead to overestimation of the neurologic complication rate. Nevertheless, the hospital admissions we analyzed are representative for Romania (18,710 admissions in 2015 for a 500-bed hospital that covers the capital city of Bucharest and 8 adjacent counties in Romania; ≈3 million inhabitants). Oseltamivir was given to all patients, often with a delay after infection onset because of late presentation. Although neuraminidase inhibitors are considered more effective against influenza A viruses than influenza B viruses, the mechanisms of influenza B virus drug resistance are not well understood (*41*). Sequencing the isolated strains was successful for isolates from 4 of the patients. Lack of amplification for the other 3 isolates could be partially associated with low levels of nucleic acids in the clinical specimens and lower sensitivity of the amplification procedures used for sequencing than the techniques used for initial virus detection.

In conclusion, we report that influenza B virus infection can cause a severe clinical course in adults, with neurologic complications in a large number of patients. Continuous evolution of influenza viruses can give rise to virulent strains that escape the immune response, particularly when a large part of the population remains unvaccinated.
